# A comparison of MEmory Specificity Training (MEST) to education and support (ES) in the treatment of recurrent depression: study protocol for a cluster randomised controlled trial

**DOI:** 10.1186/1745-6215-15-293

**Published:** 2014-07-22

**Authors:** Tim Dalgleish, Anna Bevan, Anna McKinnon, Lauren Breakwell, Viola Mueller, Isobel Chadwick, Susanne Schweizer, Caitlin Hitchcock, Peter Watson, Filip Raes, Laura Jobson, Aliza Werner-Seidler

**Affiliations:** 1 Medical Research Council Cognition and Brain Sciences Unit, 15 Chaucer Road, Cambridge CB2 7EF, UK; 2 Cambridgeshire and Peterborough NHS Foundation Trust, Cambridge Road, Fulbourn, Cambridge CB21 5HH, UK; 3 Faculty of Psychology and Educational Sciences, University of Leuven, Leuven, Tiensestraat 102, 3000 Leuven, Belgium; 4 University of East Anglia, Norwich Research Park, Norwich NR4 7TJ, UK; 5 Medical Research Council Cognition and Brain Sciences Unit, 15 Chaucer Road, CB2 7EF Cambridge, UK

**Keywords:** Depression, Memory specificity training, Autobiographical memory

## Abstract

**Background:**

Depression is a debilitating mental health problem that tends to run a chronic, recurrent course. Even when effectively treated, relapse and recurrence rates remain high. Accordingly, interventions need to focus not only on symptom reduction, but also on reducing the risk of relapse by targeting depression-related disturbances that persist into remission. We are addressing this need by investigating the efficacy, acceptability and feasibility of a MEmory Specificity Training (MEST) programme, which directly targets an enduring cognitive marker of depression - reduced autobiographical memory specificity. Promising pilot data suggest that training memory specificity ameliorates this disturbance and reduces depressive symptoms. A larger, controlled trial is now needed to examine the efficacy of MEST. This trial compares MEST to an education and support (ES) group, with an embedded mechanism study.

**Methods/Design:**

In a single blind, parallel cluster randomised controlled trial, 60 depressed individuals meeting diagnostic criteria for a current major depressive episode will be recruited from the community and clinical services. Using a block randomisation procedure, groups of 5 to 8 participants will receive five weekly sessions of MEST (n = 30) or education and support (n = 30). Participants will be assessed immediately post-treatment, and at 3- and 6-months post-treatment (MEST group only for 6-month follow-up). Depressive symptoms at 3-month follow-up will be the primary outcome. Secondary outcomes will be change in depressive status and memory specificity at post-treatment and 3-months. The 6-month follow-up of the MEST group will allow us to examine whether treatment gains are maintained. An explanatory question will examine variables mediating improvement in depression symptoms post-treatment and at 3-month follow-up.

**Discussion:**

This trial will allow us to investigate the efficacy of MEST, whether treatment gains are maintained, and the mechanisms of change. Evidence will be gathered regarding whether this treatment is feasible and acceptable as a low-intensity intervention. If efficacy can be demonstrated, the results will support MEST as a treatment for depression and provide the foundation for a definitive trial.

**Trial registration:**

NCT01882452 (ClinicalTrials.gov), registered on 18 June 2013.

## Background

Depression is a pervasive mental health condition that is a leading cause of disability worldwide [1]. As well as causing significant distress to the individual and their families, the burden of disease at the societal level is estimated to cost the United Kingdom approximately £9 billion each year [2]. Epidemiological studies have established depression as a lifelong chronic illness, with more than 80% of individuals experiencing repeated episodes [3]. Of concern is that with each successive depressive episode experienced, there is increased risk of future recurrence [4]. Gold standard psychological treatments, such as Cognitive Behavioural Therapy (CBT), can successfully treat depressive illness. However, only about half of patients receiving treatment will respond, and, even if they do, the risk of relapse and recurrence remains high [3]. If depressive disorders are to be more effectively treated in the future, a better understanding of factors that increase the risk of relapse/recurrence is needed. One way to address this need is to focus on cognitive markers of depression that do not necessarily remit following treatment.

It is widely accepted that depression is characterised by a range of disturbances in autobiographical memory. Specifically, the tendency to recall overgeneral memories (that is, memories of broader, more general categories of events rather than memories of a particular discrete instance) has been identified as a stable cognitive marker of depression [5]. Depressed individuals have difficulty retrieving specific memories of past autobiographical events in response to cue words. For example, in response to the cue word ‘excited’ there is a tendency to provide summarised categories of similar events (for example, *birthday parties*) or details of an event that occurred over a protracted time period (for example, *my holiday to Wales*), rather than a specific event (for example, *going to the Leonard Cohen concert in London last month*)*.*

Research over the past two decades has established that such overgeneral memory recall is reliably associated with depressive symptoms in both clinical and analogue samples. Furthermore, overgeneral memory recall is not just a product of being depressed - following the recovery of a depressive episode, reduced specificity remains stable. That is, formerly depressed patients show comparable levels of generality to their depressed counterparts (for example, [6,7]), suggesting that it is a trait vulnerability factor for future recurrence. In support, longitudinal findings show that the tendency to recall overgeneral memories predicts the course of depression prospectively (for example, [8-11]). Furthermore, overgeneral memory recall is associated with negative psychological outcomes that exacerbate depression including impaired problem solving ability [12], difficulty in imagining future events [13], heightened rumination [14] and executive dysfunction [15]. Therefore, this relative imbalance in the specificity of recalled autobiographical events is a key driver of depressive illness.

Theoretical accounts originally conceptualised overgenerality as a mode of memory retrieval that develops in an attempt to minimise the potential for negative emotion experience that arises from the recall of specific distressing memories [14]. This avoidance conceptualisation has since been elaborated upon, with proponents of the CaR-FA-X model (that is, capture and rumination, functional avoidance, impaired executive control) suggesting that a number of processes, either alone or in combination, account for the tendency to retrieve overgeneral memories among depressed individuals. Specifically, Williams and colleagues [5] suggest that three mechanisms underlie overgeneral memory: capture and rumination, functional avoidance and impaired executive control (for recent review, see [16]). A comprehensive analysis of this model is beyond the scope of this protocol, but in short, the capture and rumination mechanism relates to the possibility that overgeneral memory is facilitated by mnemonic information, which activates ruminative processes during memory retrieval. In support, studies have found that experimentally induced ruminative processing leads to overgeneral memory recall while non-ruminative thinking does not [17-20]. The second factor, functional avoidance, is similar to the initial conceptualisation of overgeneral memory as an avoidance mechanism, whereby remembering autobiographical events overgenerally has been suggested to reduce the affective impact of emotional memories. Consistent with this proposal are findings showing that avoiding specific recall immediately following an aversive experience reduces emotional distress [21]. Third, the model asserts that impaired executive control limits the ability of an individual to remain focused on retrieving a specific memory. To support this, numerous studies have found that overgeneral memory recall is associated with impaired executive control on a range of outcomes including verbal, spatial, and memory measures [15]. While empirical evidence is consistent with the three processes identified in the model, on-going research in the area is needed to more fully elucidate the mechanisms and interactions underlying overgeneral memory recall.

From a clinical perspective, the reliable and robust relationship between overgenerality and adverse psychological phenomena make reduced memory specificity an attractive target for therapeutic change. Moreover, research suggests that overgeneral memory recall is not a fixed feature of an individual’s mnemonic style [22] and can be modified [23]. As derived from the existing framework, it follows that enhancing access to specific memories should yield therapeutic benefit. This elegantly simple premise was first empirically addressed by Raes, Williams and Hermans [24], who developed a brief MEmory-Specificity Training (MEST) procedure. The programme was designed as a four-session, group training program whereby participants simply practiced recalling specific events repeatedly in response to different cue words. In this pilot study of 10 depressed female inpatients, results showed that memory specificity improved following the training, along with improvements in symptoms and depression-related processes (for example, rumination, problem-solving and cognitive avoidance). While promising, this first pilot included a small sample size, no control condition, and no follow-up.

Having established that in principle, memory specificity can be trained using this programme, it was followed up with a randomised clinical trial comparing MEST to a non-active control condition in a group of bereaved, depressed Afghani teenage refugees living in Iran [25]. Consistent with the earlier pilot study [24], training improved specificity levels in the MEST group. Symptom measures at post-training indicated that there was no group difference in depressive symptoms. However, participants who received MEST had significantly lower symptom levels than those in the control group at 2-month follow-up, and they were no longer in the clinical range. Finally, this improvement in depression in the MEST group was mediated by improvements in memory specificity from the beginning to the end of training.

The data from these two studies are encouraging - they suggest that MEST may improve outcomes in depression, once shifts in memory specificity have had time to gain traction in day-to-day cognitive processing. Similar encouraging data are also emerging from the post-traumatic stress disorder (PTSD) literature [26]. It is now time for the next stage of the treatment development process. Medical Research Council (MRC) guidelines [27] for the development of complex interventions specify that once the necessary pre-clinical and pilot research foundations have been completed, a Phase II exploratory trial to evaluate the efficacy, feasibility and acceptability of an intervention is needed. We are addressing this need by conducting a randomised controlled clinical trial in which MEST is compared to an appropriate, active control group.

If MEST is established as efficacious, acceptable to patients, and feasible to deliver, it would confer a number of advantages. First, it is a relatively brief and thus cost-effective intervention that may be suitable as an adjunct to more comprehensive therapies or as a standalone, low-intensity intervention for mild forms of depression. Moreover, it can be delivered by individuals with limited training and experience, and, as it does not involve addressing emotional difficulties, lends itself to implementation in challenging clinical contexts by individuals who do not necessary have the training in how to manage sensitive issues that arise in clinical groups. This is particularly important in countries with fewer mental-health resources [28]. From a public health perspective, this treatment is very much in line with UK government initiatives aiming to deliver more accessible, cost-effective psychosocial interventions, such as the Improving Access to Psychological Therapies (IAPT) programme [29]. Another advantage associated with MEST is that it targets a cognitive marker of depression that does not necessarily improve as an individual comes out of a depressive episode (for example, [8,30]). This provides reason to expect that this intervention may reduce future relapse and recurrence via its impact on specificity-related processes.

A key question that remains unanswered is exactly *how* MEST works. It is hypothesised that MEST training changes processes believed to mediate the impact of the mode of memory retrieval on depression, namely, by decreasing cognitive avoidance, reducing rumination, improving problem solving, and improving executive control. However, the degree to which changes in these variables is mediated by changes in memory specificity is unknown. An embedded mechanism study will allow investigation of the processes mediating change.

In summary, there is promising evidence suggesting that MEST offers a novel way through which a key memory disturbance linked to depression and adverse psychological sequelae may be targeted. In this cluster RCT we will compare MEST to an active education and support (ES) intervention control condition in a sample of depressed British adults. This trial was designed to answer four key questions. First, does MEST improve specificity and reduce depression? Second, are gains maintained over the longer term? Third, is MEST superior to an education and support intervention in its effects on depression and specificity? Fourth, what are the mechanisms mediating any treatment effects?

## Methods/Design

### Study design

The design is a single-blind, parallel cluster RCT comparing MEST to an ES intervention, with an embedded mechanism study. The intervention duration is equivalent in both conditions and participants will be assessed four times - at baseline, at post-treatment, and at 3-month and 6-month follow-up time points. The first three assessment points (that is, baseline, post, and 3-month follow-up) are face-to-face and include the full battery of assessment measures, including primary and secondary outcomes and process measures. Assessment at 6 months will be for the MEST group only and will take place via phone. This final assessment includes primary and secondary outcomes only (with the exception of the Autobiographical Memory Test; AMT).

### Participants and recruitment

A total of sixty individuals aged 18 to 65 with a principle diagnosis of major depressive disorder (recurrent episode) will be randomised to either MEST (*n* = 30) or ES (*n* = 30). Inclusion criteria are a diagnosis of major depressive disorder (recurrent) according to the DSM-V; a diagnosis of a current major depressive episode; the presence of moderate symptoms defined as a Beck Depression Inventory (BDI-II) [31] score of >13, and memory specificity of less than 0.70, as assessed on the AMT [32]. Participants need to have a baseline level of specificity of less than 0.70 to be invited into the trial because a higher level illustrates that memory specificity is not a difficulty for that individual, and there will be minimal opportunity for specificity-related benefits to accrue. Participants continue with their usual treatment (for example, antidepressant medication or psychological therapy) during the trial. Exclusion criteria are: high levels of suicidality or harm to others (assessed via interview and exclusion according to clinical judgement), a secondary diagnosis of another affective disorder or a psychotic disorder; current drug/alcohol abuse or dependence all assessed via the Structured Clinical Interview for the Diagnostics and Statistics Manual – SCID-V, [33]; a diagnosed Axis II disorder (assessed via participant report); and the presence of head trauma or organic brain damage (assessed via participant report).

There are three pathways through which participants will be recruited. The first is directly from the community via posters and advertisements around the Cambridge (UK) area. The second is from our departmental volunteer panel comprising individuals with a history of depression who have previously participated in our research. The third will be via clinical services offered within the Cambridgeshire and Peterborough Foundation Trust. Patients in these services will be identified by members of the clinical team and provided information about the trial. They will be able to opt in by contacting us directly.

Participants who have previously been assessed by our research team will be notified about the study by email. Participants who have not previously volunteered in our research (that is, community members and patients in clinical services) who express interest will be provided written information about the study (via email or post) after contacting us. After reading the study information sheet, interested participants will be administered a brief phone screen (using items from the Mood Module of the SCID-IV) to verify whether they meet criteria for a current major depressive episode. Those who do will then be invited to attend a session at the Medical Research Council Cognition and Brian Sciences Unit for a full baseline assessment involving clinical interviews and experimental tasks, as outlined below.

### Participant allocation

**Figure 1:**
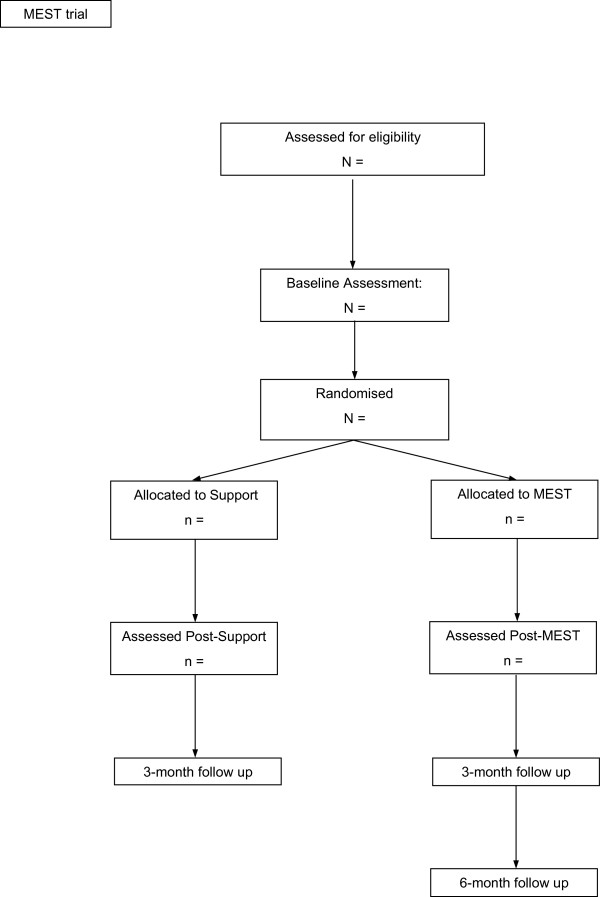
CONSORT diagram.

Eligible participants will be cluster randomised using a blocked randomisation procedure with 5 to 8 participants in a group to receive the intervention. This will be achieved using computer-generated, quasi-random numbers and will be conducted by the trial statistician (Watson), blind to study objectives. Once generated, this information is passed to the trial lead (Dalgleish), who informs the project coordinator responsible for delivering the intervention (Werner-Seidler) within 48-hours of the commencement of a given group.Figure 1 summarises the trial CONSORT (consolidated standards for reporting trials) diagram. Following randomisation, patients are offered treatment for five consecutive weeks (commencing within four weeks of the baseline assessment).

### Interventions

#### MEmory Specificity Training

MEST is the training package developed by Raes and colleagues, used in the first pilot study of memory specificity training [24]. For the current study, the original treatment manual was translated from Dutch into English, and, in collaboration with the treatment developer, has been extended from four to five sessions, as used in our pilot RCT [25]. It consists of a fully manualised, structured treatment delivered over five 60-minute sessions to groups of 5 to 8 individuals. The treatment aims to enhance memory specificity through the systematic practice of memory retrieval in response to emotional and neutral cue words. This practice takes place both during the sessions and at home.

The first session involves providing basic psycho-education about depressive symptoms and memory difficulties common in depression. As a group, participants then practice recalling memories in response to positive and neutral cues, with demonstrations and support from the therapists. For homework, participants have to identify specific memories in response to ten cue words, and identify an additional ‘memory of the day’ each day. The second session follows the same format with further practice. For homework, participants must identify specific memories in response to ten cue words, and identify two further daily specific memories. In the third session, practice in response to negative cues (as well as positive cues) is introduced, and homework is identical to that following Session 2. Session 4 involves practice to positive, negative, and neutral cues and also includes an example of noticing overgeneral thinking in everyday contexts. For homework, participants identify memories in response to ten cues, identify a single ‘memory of the day’ each day, and provide a daily example of when they have noticed themselves thinking in an overgeneral fashion. The final session involves further practice and revision of the material covered throughout the treatment.

#### Education and support

The ES intervention will follow the same format and length as the MEST intervention (that is, delivered to groups of 5 to 8 individuals over five 60-minute sessions). ES has been manualised for this trial and is a cognitively-orientated intervention. The aim is to provide a non-judgemental, supportive environment in which participants are encouraged to raise and discuss a range of events that occur each week. These events may be positive, negative, or benign and non-emotional in nature: it is up the individuals to bring to the group whatever they would like. Through reflection and discussion, the goal is to support and help individuals attain optimal adjustment and to better cope with everyday life. This approach draws on the supportive aspects of the therapeutic relationship to assist with more effective psychological functioning, with input from other group members. It is a non-directive intervention whereby therapists do not use directive methods such as cognitive-behavioural techniques (for example, Socratic questioning, cognitive restructuring, behavioural experimentation).

The rationale for choosing an education and support comparison group over more traditional psychological approaches (for example, supportive counselling, CBT) was to ensure that the content covered by the two interventions is as similar as possible. More traditional approaches tend to focus on distressing and emotional material, which is not the focus of the MEST intervention. Therefore, to optimise equivalence in content between the groups we decided to take a cognitive approach and ask people to record material from each week that can be positive, negative or neutral. The role of the group is to then help individuals to notice patterns in their responses and adopt a self-reflective approach, which may improve psychological functioning.

In the first session, psycho-education about depressive symptoms is presented and the experience of group members is normalised. Participants are invited to share why they have decided to participate in this study. An outline of the programme is then provided and participants are informed about how to complete the homework daily diary where they note down a significant event that occurred each day, which can be shared in the following session. The second session involves reviewing the homework and continued discussion of events that participants have noted down in their diaries. These discussions are led by the therapists, but as with the MEST intervention, input from other group members is encouraged. Sessions 3, 4, and 5, follow an identical structure - a homework review, followed by discussion and support, with the homework task to record a positive, negative or neutral daily event for discussion at the next session. The final session does not require completion of homework tasks. The ES manual was developed by two clinical psychologists (Werner-Seidler and Dalgleish), in consultation with two experienced counsellors from the Cambridge area.

#### Treatment integrity

Two clinical psychologists with experience in treating adult depression will deliver the MEST and ES interventions. Treatment fidelity and clinician adherence will be established using continued monitoring and through independent rating. After every session, clinicians will complete the Treatment Fidelity Checklist, which is a session-by-session measure of compliance with the protocol, and these will be evaluated during weekly clinical supervision with the trial lead. In addition, a random 25% of the audio-taped treatment sessions will be rated for adherence to the manuals by an experienced clinician, independent of the core trial team. Homework completion will be monitored and assessed using self-report questionnaire measures.

### Measures

#### Primary outcomes

The primary outcome measure will be depressive symptoms at 3-month follow-up using the well-established, psychometrically sound self-report measure, the Beck Depression Inventory II (BDI-II) [31].

#### Secondary outcomes

Secondary outcomes will be depressive diagnostic status and depression-free days since the beginning of treatment at the post, 3-month and 6-month (MEST group only) follow-up assessment as measured by the Longitudinal Interval Follow-up Evaluation (a form of the Structured Clinical Interview for DSM-V, which is the gold standard for diagnostic assessment) [33]; depressive symptoms at post-treatment and 6-month follow-up (as measured by the BDI-II); and memory specificity at post and 3-month follow-up, as measured by the gold standard in memory specificity research, the AMT [32].

#### Process measures

To address our explanatory question concerning the mechanisms by which MEST improves outcomes in depression, we include a number of process-related tasks, parallel forms of which will be administered at baseline, at post-intervention, and at 3-month follow-up, across the trial arms. Accordingly, change on theoretically-derived processes from pre- to post-treatment (outlined below) will be used to predict treatment outcome at follow-up using mediational analyses [34]. Derived from theory and evidence, we will examine whether the effect of MEST on treatment outcome is mediated by changes in memory specificity, interpersonal problem solving, rumination, cognitive avoidance, and executive control. Problem-solving will be measured using a shortened version of the Means-Ends Problem Solving task [35], which has been used previously in clinical research. Rumination and cognitive avoidance will be measured using the psychometrically valid gold-standard self-report measures for each construct - the Rumination Response Scale (RRS) [36] and the Cognitive Avoidance Questionnaire [37]. Executive control will be indexed via verbal fluency and working memory measures commonly used in neuropsychological assessment [38].

### Methodological aspects

#### Power analysis and sample size

Although a standard power calculation based on detecting treatment effects would be the conventional approach to determining sample sizes for clinical trials, the main aim of the current exploratory trial is to investigate the efficacy, acceptability and feasibility of MEST, in preparation for scaled up later phase evaluations of the intervention in line with MRC guidance. Our previous experience with Phase II exploratory trial platforms indicates that 60 participants (30 in each arm) will provide sufficient numbers to evaluate outcome, feasibility and acceptability, and to generate preliminary process data for this treatment. This sample size will give 24 patients in each arm if we allow for 20% attrition. This will provide a plausible range of point estimates of effect on our set of outcome measures sufficient to guide us in sample size calculations for later phase trial work.

#### Data collection

Outcome data will be collected on site at three assessment points (baseline, post-treatment, and 3-month follow-up). Outcome data on all participants who are randomised will be collected if possible and with appropriate consent. At 6-month follow-up, participants from the MEST arm will be contacted by phone and administered the Longitudinal Interval Follow-Up Evaluation to ascertain depression-free days (a form of the SCID-IV), and will be asked to complete the BDI-II electronically. Memory specificity and the full process battery will not be administered at the 6-month follow-up. Data will be stored securely at this site under the management of the trial coordinator (Werner-Seidler). Spot checks on data entry will be performed to promote data quality.

#### Blinding

Outcome assessments are conducted by independent raters who have no therapeutic relationship with the patients and are blind to treatment condition. Double blinding of patients and therapists is not possible due to the nature of the trial (that is, a psychological intervention). Under no circumstance will unblinding be necessary since participants and therapists are not blinded to intervention allocation.

#### Statistical analysis plan

Initial analyses of the primary and secondary outcomes will be conducted by the trial statistician, following CONSORT standards (there are no planned interim analyses). Multiple imputation will be used to handle missing data. Initial analyses will be conducted on an intention-to-treat basis, with subsequent analyses being per protocol. Mixed model analyses of variance (ANOVAs) will be used to compare groups at the four assessment points - baseline, post-intervention, 3-month follow-up, and 6-month follow-up on primary and secondary outcomes, as appropriate. Baseline levels on relevant measures will be included as covariates, as appropriate. Mediational analyses following the Kraemer recommendations [34] will investigate hypothesised mechanisms of change across the trial arms.

#### Monitoring and data management

The trial will take place at the Medical Research Council’s Cognition and Brain Sciences Unit in Cambridge, England. As a Phase II trial, a data-management committee was considered unnecessary and the trial team is therefore responsible for monitoring and data management. Data will be monitored for completeness, consistency, and plausibility using spot checks and plausibility checks carried out by the trial statistician. The trial lead (Dalgleish), statistician (Watson) and coordinator (Werner-Seidler) will have full access to the final trial dataset. The study data will be reported in line with the current CONSORT recommendations [39].

#### Safety aspects

Adverse events refer to unwanted medical events (for example, worsening symptoms) occurring throughout the trial, regardless of whether they are causally related to the trial procedures. Adverse events are managed in line with UK MRC protocols, and in the unlikely case of an adverse event, it will be documented appropriately. Precautions have been taken to reduce the likelihood of adverse events occurring; for example, patients who are acutely suicidal or at high risk of harm do not meet study inclusion criteria. The interventions are delivered by clinical psychologists experienced in the management of risk and in the treatment of chronic, recurrent, and severe depression. In the case of any adverse events, participation in the trial will be discontinued. The trial is underwritten by the UK MRC in case any individual suffers harm or requires post-trial care.

#### Ethical approval and protocol amendments

This project has received ethical approval from the UK National Research Ethics Committee (East of England, 11/H0305/1). The study will be conducted within appropriate UK MRC, National Health Service and professional ethical guidelines, ensuring that Good Clinical Practice procedures are adhered to at all times. Protocol amendments will be circulated to the ethics, research and development, and trial team. Relevant adjustments will be made to any published protocol.

#### Confidentiality

All participants will give written informed consent prior to being assessed for eligibility of the study. To maintain confidentiality, all participants are given a trial number so that personally identifying information is not linked to assessment or trial information.

#### Dissemination policy

There are no publication restrictions, and findings will be disseminated broadly to participants, healthcare professionals, the public, and other relevant groups.

## Discussion

According to the World Health Organisation [1], depression is major global burden of disease and, by 2020, will be the second leading cause of world disability. Depression affects people in all communities across the world, making it a matter of global concern from health, social and economic perspectives. Fortunately, evidence-based treatments that work such as cognitive-behavioural therapies are available. However, these treatments are not always accessible to those who need them, and the treatments themselves are expensive to deliver. There has been a growing recognition of the need to develop cost-effective interventions that can be delivered in a stepped care model, supported by a number of government initiatives (particularly in the UK). The underlying goal is to make evidence-based interventions more accessible and affordable (for example, NHS internet-based CBT, IAPT). The proposed RCT is in line with this objective.

The two pilot trials that have been conducted suggest that MEST, if efficacious in the treatment of depression, may offer a cost-effective, brief, low-intensity intervention with potential for cross-cultural application. For example, one of the pilot studies was conducted with Afghan refugees in Iran, the other in an inpatient sample in Belgium. This is of particular interest given that risk factors for depression include economic and social disadvantage [40] and those in impoverished communities have greater difficulty accessing health care [41]. If acceptability, feasibility, and superiority to the ES control can be demonstrated, the results from this trial will support the need for a fully powered Phase III definitive trial to examine MEST as a treatment for mild to moderately depressed individuals. We anticipate that MEST may be used as an adjunct or precursor to more comprehensive therapies such as CBT. It is possible that MEST may have utility in reducing symptoms to a level where patients are able to engage in more demanding therapies such as CBT. Results of this trial will also indicate whether there is potential for MEST to be used as a stand-alone treatment for depression. Further, we expect that the idiosyncratic nature of MEST may appeal to particular patient groups who do not wish to address emotional content. Finally, the embedded process study will enable us to better understand the mechanisms driving the therapeutic effects of the treatment.

## Trial status

Recruitment commenced in January 2014.

## Abbreviations

AMT: Autobiographical Memory Test; BDI-II: Beck Depression Inventory-II; CBT: cognitive behavioural therapy; CONSORT: consolidated standards for reporting trials; DSM: Diagnostic and Statistical Manual; ES: education and support; IAPT: Improving Access to Psychological Therapies; MEST: Memory Specificity Training; MRC: Medical Research Council; NHS: National Health Service; PTSD: post-traumatic stress disorder; RCT: randomised controlled trial; SCID-IV: Structured Clinical Interview for DSM-IV disorders.

## Competing interests

The authors declare that they have no competing interests.

## Authors’ contributions

TD designed the study, developed and adapted the treatment manuals, and helped to draft the manuscript; AB delivered the interventions; AM delivered the interventions; LB helped coordinate participant recruitment, conducted blind assessments and was involved in data entry; VM conducted blind assessments; IC conducted blind assessments; SS translated the manual from Dutch to English; CH assisted in coordinating the project; PW performed the randomisation, advised on statistical approach and will analyse the data; FR advised on the delivery of the interventions and provided guidance on the protocol; LJ performed blind ratings of interviews and assessment; and AWS developed and adapted the treatment manuals, coordinated recruitment, conducted baseline assessments, delivered the interventions and drafted the manuscript. All authors read and approved the final manuscript.

## Authors’ information

All authors are affiliated with the Medical Research Council, Cognition and Brain Sciences Unit, except FR, who is affiliated with the Faculty of Psychology and Educational Sciences, University of Leuven, and LJ, who has a joint affiliation between Medical Research Council, Cognition and Brain Sciences Unit and the University of East Anglia, Norwich, UK. TD, AB, AM and AW-S have an additional affiliation with the Cambridgeshire and Peterborough NHS Foundation Trust.
